# Supplementary material for: Multi‐dataset identification of innovative feature genes and molecular mechanisms in keratoconus
Source: J Cell Mol Med. 2024 Sep 19;28(18):e70079. doi: 10.1111/jcmm.70079 (PMC11412914; doi:10.1111/jcmm.70079)
Supplement: Supplementary file 2 — Material S2. [file JCMM-28-e70079-s002.zip › 7.GSEA analysis/3.DBNDD1/KEGG_ADIPOCYTOKINE_SIGNALING_PATHWAY.html]

Details for gene set KEGG\_ADIPOCYTOKINE\_SIGNALING\_PATHWAY[GSEA]

|  || Dataset | DBNDD1.group.cls#h\_versus\_l |
| Phenotype | group.cls#h\_versus\_l |
| Upregulated in class | l |
| GeneSet | KEGG\_ADIPOCYTOKINE\_SIGNALING\_PATHWAY |
| Enrichment Score (ES) | -0.32737413 |
| Normalized Enrichment Score (NES) | -1.1732697 |
| Nominal p-value | 0.27831095 |
| FDR q-value | 0.7102656 |
| FWER p-Value | 0.991 |
Table: GSEA Results Summary

  

Fig 1: Enrichment plot: KEGG\_ADIPOCYTOKINE\_SIGNALING\_PATHWAY      
 Profile of the Running ES Score & Positions of GeneSet Members on the Rank Ordered List

  

| SYMBOL | TITLE | RANK IN GENE LIST | RANK METRIC SCORE | RUNNING ES | CORE ENRICHMENT || 1 | ADIPOQ | na | 1141 | 0.368 | 0.0248 | No |
| 2 | CAMKK1 | na | 1922 | 0.305 | 0.0501 | No |
| 3 | PPARGC1A | na | 2929 | 0.255 | 0.0610 | No |
| 4 | LEP | na | 6081 | 0.148 | -0.0067 | No |
| 5 | PRKCQ | na | 6488 | 0.137 | 0.0031 | No |
| 6 | IRS4 | na | 7914 | 0.105 | -0.0216 | No |
| 7 | AKT2 | na | 9388 | 0.080 | -0.0515 | No |
| 8 | PCK1 | na | 9869 | 0.073 | -0.0539 | No |
| 9 | CAMKK2 | na | 10429 | 0.065 | -0.0598 | No |
| 10 | MAPK10 | na | 10497 | 0.065 | -0.0516 | No |
| 11 | MAPK9 | na | 11537 | 0.052 | -0.0735 | No |
| 12 | RXRA | na | 12017 | 0.047 | -0.0799 | No |
| 13 | ACSL1 | na | 12343 | 0.044 | -0.0824 | No |
| 14 | AGRP | na | 12417 | 0.043 | -0.0778 | No |
| 15 | MTOR | na | 13075 | 0.038 | -0.0908 | No |
| 16 | STK11 | na | 13395 | 0.035 | -0.0945 | No |
| 17 | CPT1A | na | 13804 | 0.032 | -0.1014 | No |
| 18 | PRKAG1 | na | 14780 | 0.024 | -0.1257 | No |
| 19 | PPARA | na | 15489 | 0.019 | -0.1431 | No |
| 20 | ADIPOR1 | na | 15720 | 0.018 | -0.1469 | No |
| 21 | PRKAG3 | na | 16144 | 0.015 | -0.1568 | No |
| 22 | PRKAB1 | na | 16608 | 0.012 | -0.1683 | No |
| 23 | TRADD | na | 16696 | 0.011 | -0.1690 | No |
| 24 | PRKAG2 | na | 17777 | 0.004 | -0.1995 | No |
| 25 | ACACB | na | 18147 | 0.002 | -0.2099 | No |
| 26 | POMC | na | 18926 | -0.004 | -0.2317 | No |
| 27 | CHUK | na | 18953 | -0.004 | -0.2318 | No |
| 28 | AKT1 | na | 19066 | -0.005 | -0.2343 | No |
| 29 | PTPN11 | na | 19596 | -0.009 | -0.2480 | No |
| 30 | PRKAA1 | na | 20353 | -0.016 | -0.2673 | No |
| 31 | ADIPOR2 | na | 20495 | -0.017 | -0.2687 | No |
| 32 | JAK2 | na | 21143 | -0.023 | -0.2837 | No |
| 33 | RXRB | na | 21455 | -0.026 | -0.2885 | No |
| 34 | NFKBIB | na | 21812 | -0.030 | -0.2941 | No |
| 35 | TRAF2 | na | 22056 | -0.033 | -0.2960 | No |
| 36 | IKBKB | na | 22300 | -0.035 | -0.2975 | No |
| 37 | MAPK8 | na | 22620 | -0.039 | -0.3007 | No |
| 38 | PRKAB2 | na | 22826 | -0.041 | -0.3001 | No |
| 39 | TNFRSF1A | na | 22847 | -0.041 | -0.2943 | No |
| 40 | IKBKG | na | 23063 | -0.044 | -0.2936 | No |
| 41 | ACSL3 | na | 24235 | -0.059 | -0.3181 | Yes |
| 42 | CD36 | na | 24242 | -0.060 | -0.3089 | Yes |
| 43 | ACSL5 | na | 24724 | -0.067 | -0.3123 | Yes |
| 44 | PRKAA2 | na | 24976 | -0.070 | -0.3085 | Yes |
| 45 | CPT1C | na | 25125 | -0.073 | -0.3014 | Yes |
| 46 | ACSL4 | na | 25273 | -0.075 | -0.2939 | Yes |
| 47 | STAT3 | na | 25462 | -0.078 | -0.2872 | Yes |
| 48 | NFKB1 | na | 25842 | -0.084 | -0.2849 | Yes |
| 49 | SLC2A4 | na | 26020 | -0.088 | -0.2762 | Yes |
| 50 | G6PC2 | na | 26032 | -0.088 | -0.2627 | Yes |
| 51 | SLC2A1 | na | 26795 | -0.103 | -0.2685 | Yes |
| 52 | TNF | na | 27164 | -0.111 | -0.2617 | Yes |
| 53 | CPT1B | na | 27252 | -0.114 | -0.2464 | Yes |
| 54 | NFKBIA | na | 27480 | -0.118 | -0.2344 | Yes |
| 55 | RXRG | na | 28564 | -0.145 | -0.2429 | Yes |
| 56 | RELA | na | 28616 | -0.147 | -0.2214 | Yes |
| 57 | LEPR | na | 29224 | -0.163 | -0.2133 | Yes |
| 58 | TNFRSF1B | na | 29728 | -0.180 | -0.1997 | Yes |
| 59 | NFKBIE | na | 29778 | -0.182 | -0.1726 | Yes |
| 60 | ACSL6 | na | 30844 | -0.216 | -0.1696 | Yes |
| 61 | IRS2 | na | 31498 | -0.240 | -0.1508 | Yes |
| 62 | SOCS3 | na | 31574 | -0.244 | -0.1148 | Yes |
| 63 | PCK2 | na | 32336 | -0.284 | -0.0923 | Yes |
| 64 | NPY | na | 32366 | -0.286 | -0.0483 | Yes |
| 65 | IRS1 | na | 33372 | -0.358 | -0.0212 | Yes |
| 66 | AKT3 | na | 33656 | -0.385 | 0.0309 | Yes |
Table: GSEA details [plain text format]

  

Fig 2: KEGG\_ADIPOCYTOKINE\_SIGNALING\_PATHWAY      
 Blue-Pink O' Gram in the Space of the Analyzed GeneSet

  

Fig 3: KEGG\_ADIPOCYTOKINE\_SIGNALING\_PATHWAY: Random ES distribution      
 Gene set null distribution of ES for **KEGG\_ADIPOCYTOKINE\_SIGNALING\_PATHWAY**
